# Mandibular Third Molar Surgery: Intraosseous Localization of the Inferior Alveolar Nerve Using 3D Double-Echo Steady-State MRI (3D-DESS)

**DOI:** 10.3390/diagnostics11071245

**Published:** 2021-07-12

**Authors:** Adib Al-Haj Husain, Bernd Stadlinger, Sebastian Winklhofer, Marcel Müller, Marco Piccirelli, Silvio Valdec

**Affiliations:** 1 Center of Dental Medicine, Clinic of Cranio-Maxillofacial and Oral Surgery, University of Zurich, 8032 Zurich, Switzerland; adib.alhaj@gmail.com (A.A.-H.H.); bernd.stadlinger@zzm.uzh.ch (B.S.); 2 Clinical Neuroscience Center, Department of Neuroradiology, University Hospital of Zurich, University of Zurich, 8091 Zurich, Switzerland; sebastian.winklhofer@usz.ch (S.W.); marco.piccirelli@usz.ch (M.P.); 3 Statistical Services, Center of Dental Medicine, University of Zurich, 8032 Zurich, Switzerland; marcel.mueller@zzm.uzh.ch; 4 Department of Stomatology, Division of Periodontology, Dental School, University of São Paulo, Butantã 2227, SP, Brazil

**Keywords:** magnetic resonance imaging, inferior alveolar nerve, oral surgery, radiology, anatomy

## Abstract

The aim of this study was to evaluate the inferior alveolar nerve’s (IAN) intraosseous position within the inferior alveolar canal (IAC) using a 3D double-echo steady-state MRI sequence (3D-DESS). The IAN position was prospectively evaluated in 19 patients undergoing mandibular third molar (MTM) surgery. In the coronal reference layer, the IAC was divided into six segments. These segments were checked for the presence of hyperintense tubular MRI signals representing the IAN’s nervous tissue and assessed as visible/non-visible. Furthermore, the IAN in MRI and the IAC in MRI and CBCT were segmented at the third and second molar, determining the maximum diameter in all planes and a conversion factor between the imaging modalities. Regardless of the positional relationship at the third and second molar, the IAN showed the highest localization probability in the central segments (segment 2: 97.4% vs. 94.4%, segment 5: 100% vs. 91.6%). The conversion factors from IAC in CBCT and MRI to IAN in MRI, respectively, were the following: axial (2.04 ± 1.95, 2.37 ± 2.41), sagittal (1.86 ± 0.96, 1.76 ± 0.74), and coronal (1.26 ± 0.39, 1.37 ± 0.25). This radiation-free imaging modality, demonstrating good feasibility of accurate visualization of nervous tissue within the nerve canal’s osseous boundaries, may benefit preoperative assessment before complex surgical procedures are performed near the IAC.

## 1. Introduction

Accurate preoperative visualization of the inferior alveolar nerve (IAN), the largest branch of the mandibular nerve located within the inferior alveolar canal (IAC), is of clinical interest to minimize the risk of nerve injury during various dentoalveolar surgical interventions such as orthognathic surgery [1], dental implant insertion [2], mandibular block analgesia [3], and surgical extraction of mandibular third molars (MTM) [4].

Iatrogenic injury to the IAN resulting from third molar surgery leads to sensory disorders, ranging from partial loss of sensitivity to complete neurosensory loss [5]. The frequency of these neurosensory disorders is reported in the literature to be approximately 4% (0.4%–8.4%) [5,6]. In case of nerve damage, there is a wide range of possible complaints reported by affected patients such as altered sensation, painful sensation, or even complete loss of sensation in the area supplied by the IAN. Besides additional neuropathic pain, dysesthesia, hyperalgesia, or paresthesia may occur [7]. In most cases, full recovery occurs within the first 6 to 8 weeks postoperatively; if not, the probability of permanent neurosensory deficiency increases remarkably [8]. This can lead to a loss of quality of life, often accompanied by psychological and social complaints [9].

Consequently, precise preoperative clarification of the IAN’s intraosseous anatomy by three-dimensional imaging is of clinical significance to reduce the risk in surgical procedures performed nearby the IAC. The routine modality of choice is the two-dimensional imaging by panoramic radiography (PAN) regarding MTM surgery [10]. In various cases, where anatomical structures are superimposed, PAN is not sufficient, and a three-dimensional imaging modality is indicated. This concerns cases where the roots of the third molars have a close relationship to the IAC with at least darkening of one root, a non-continuous cortical line of the IAC, or diversion of the IAC [11]. For this purpose, cone-beam computed tomography (CBCT) is the “gold standard” in dental clinical routine, allowing for optimal visualization of the relationship between the third molar and the opacities of the osseous cortical boundaries of the IAC. This imaging modality’s general disadvantages are the high radiation exposure, especially for the thyroid [12], and the globally reduced accessibility [13]. Although CBCT, compared to the alternative traditional computed tomography (CT), has deficiencies in soft tissue contrast and uniform grayscale values, it is the preferred choice in dental imaging due to its lower radiation exposure, lower costs, and higher availability [14]. Conventional X-ray-based radiation imaging modalities such as PAN, CBCT, or CT can visualize the osseous boundaries of the IAC, whereas the nerve itself cannot be displayed. For this purpose, magnetic resonance imaging (MRI), providing radiation-free dental imaging, is a suitable tool [15]. MRI enables excellent soft tissue contrast and has established itself as one of the leading imaging modalities in the head and neck region, despite having some limitations regarding hard tissue contrast. The increasing number of MRI studies in the context of dental treatments confirms the importance and perspectives that are opening up due to the targeted use of certain aids, such as intraoral coils [16] or radiofrequency (RF) coils [17]. A difficulty in oral cavity MRI might be the reduced image quality due to movement artifacts, field inhomogeneity, implants, or metallic dental restorations [18,19].

Since the IAN’s intraosseous course cannot be displayed by conventional radiographic assessment, an increasing number of MRI studies have investigated its direct visualization, with only a few studies achieving promising reliable results in the context of third molar surgery [20,21]. Excellent visualization of the IAN was enabled by applying the recently introduced 3D double-echo steady-state (3D-DESS) with water excitation MRI sequence [22,23,24,25]. Previous reports documenting the IAN’s intraosseous course had various significant limitations, such as using CBCT imaging displaying only the nerve canal [26] or skull studies using cadavers not allowing for generalized information or having edentulous mandibles [27,28].

This anatomical study aimed to evaluate the direct visualization of the IAN’s intraosseous localization at the level of the third molar using 3D-DESS MRI and simultaneously assessing a conversion factor for the axial, sagittal, and coronal planes between the IAC in the CBCT images and the IAN in the 3D-DESS MR images in complex clinical situations where preoperative 3D diagnostics using CBCT are indicated based on objective criteria.

## 2. Materials and Methods

### 2.1. Study Design

This prospective cohort study included 23 patients, recruited between May 2018 and December 2018, with an indication for removal of retained or impacted MTMs with a positional relationship to the IAN, indicating three-dimensional imaging according to the guiding principles of the Swiss association of dentomaxillofacial radiology. All study participants underwent CBCT and MRI scans preoperatively. Four volunteers did not show up for MRI or CBCT imaging procedures; therefore, 36 inferior alveolar nerves were evaluated (19 patients, 19 nerves on each side; in two cases, the MTM was missing). The study population enrolled patients admitted to the Clinic of Cranio-Maxillofacial and Oral Surgery of the Center of Dental Medicine (University of Zurich) by referral through a private practitioner or by themselves. MRI data acquisitions were performed by trained neuroradiologists, while CBCT data acquisitions were carried out by trained research personnel of the Clinic of Cranio-Maxillofacial and Oral Surgery. Oral surgeons subsequently performed the MTM surgery. The sex ratio was 6 males (32%) to 13 females (68%), and the mean age was 30.5 ± 13 years (median age, 25 years; age range, 18–63 years) (Table 1). Based on the same dataset, the preoperative visualization of the lingual nerve was assessed and will be published separately.

The inclusion criteria were as follows: age between 18 to 65 years and indication for impacted MTM surgery. Exclusion criteria were acute odontogenic infection, nerve damage to the three large branches of the trigeminal nerve (ophthalmic branch (V_1_), maxillary branch (V_2_), and mandibular branch (V_3_)), adjacent implants or metallic reconstructions, and the common contraindication for MRI imaging such as pregnancy, metallic intraocular foreign bodies, cerebral aneurysm clips in the brain and cardiovascular implantable electronic devices.

The study (BASEC-Nr. 2017-01053) received ethical approval from the Cantonal Ethics Commission of Zurich (Switzerland). Before performing the experiments, all volunteers were informed and provided written informed consent in accordance with the 1964 Helsinki declaration and its later revised ethical standards. Additionally, this study complies with the “Strengthening the Reporting of Observational studies in Epidemiology” (STROBE) guidelines.

### 2.2. MRI Data Acquisition

All study participants underwent MRI on a 3 Tesla Skyra (release VE11c, Siemens Healthineers, Erlangen, Germany) using a Siemens standard 64 channel head-and-neck coil. The used axial 3D-DESS MRI sequence had an isotropic acquisition resolution of 0.75 × 0.75 × 0.75 mm^3^ together with a receive bandwidth of 355 Hz/Px. The other sequence specifications were field-of-view 242 × 242 × 78 mm^3^, acquisition matrix 320 × 320 × 104, slice oversampling 100%, no parallel acquisition, one signal average, acquisition time 12:24 min:s, TR/TE1/TE2 11.2/4.2/7.7 ms, flip angle 30°, and selective water excitation.

### 2.3. CBCT Data Acquisition

The CBCT images were acquired using the Orthophos SL 3D scanner (Dentsply Sirona, Bensheim, Germany). The positioning lasers of the scanner were used to position the head of each participant. Additionally, adjusted head supports and chin rests were used during CBCT scanning time. The CBCT standard protocol was applied containing these specifications: 85 kV, 13 mA, radiation time 4.4 s, voxel size 160 μm, and FOV 11 × 10 cm.

### 2.4. Image Evaluation

The CBCT and 3D-DESS MRI DICOM data were stored and analyzed in the local Picture Archiving and Communication System (PACS) (IMPAX EE R20, release XV, Agfa Healthcare, Mortsel, Belgium) using a 2-megapixel high-resolution liquid-crystal display. The coronal MRI layer showing the closest positional relationship between the IAN and the MTM was selected as the reference image to determine the precise intraosseous anatomy of the IAN. The area of the IAC, determined by the osseous boundaries, was divided into six segments. These segments were checked for the presence of a hyperintense tubular signal representing the IAN’s nervous tissue. The same evaluation was performed at the second molar’s distal root to examine the IAN’s intraosseous position outside the third molar region. Quantitative analysis of the reference images at the level of the third molar and the distal root of the second molar was performed by using semi-automated PACS contour segmentation of the IAC in the CBCT and the IAC and IAN in the 3D-DESS MRI images, with which the maximum diameter in the coronal, axial, and sagittal planes was determined. Generating this information, a conversion factor between the two imaging modalities CBCT and MRI for each plane was determined.

#### 2.4.1. Qualitative Readout

**Figure 1:**
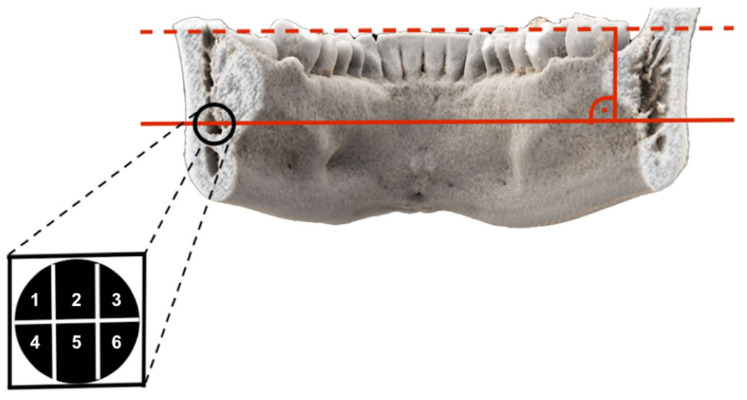
Photorealistic three-dimensional (3D) visualization of a study participant’s cone-beam computed tomography (CBCT) using cinematic rendering (CR). The coronal layer with the closest positional relationship between the inferior alveolar nerve (IAN) and the third molar is visualized. A horizontal line was placed on the selected reference layer through the alveolar crest of the buccal cortical plate at the height of the third molar on the left and right sides. A parallel line was placed through the midpoint of the IAN, whereby the section representing the intraosseous diameter of the inferior alveolar canal (IAC) was divided by two perpendiculars, resulting in six segments: segment one, upper buccal segment; segment two, upper middle segment; segment three, upper lingual segment; segment four, lower buccal segment; segment five, lower middle segment; and segment six, lower lingual segment.

**Figure 2:**
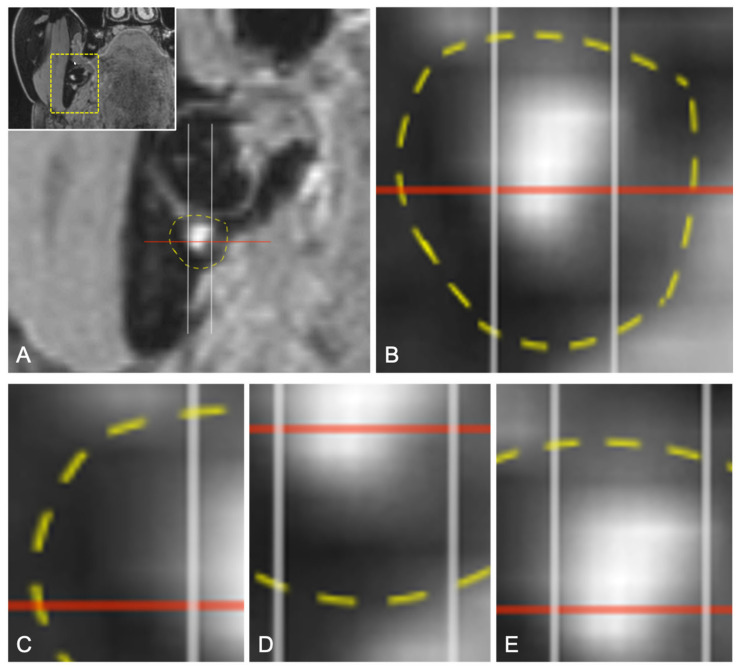
(**A**) Enlarged coronal reconstruction of the 3D-DESS images showing the mandibular third molar and the IAN (MRI signal hyperintensities) with its diameter (red line) located within the IAC (yellow line). (**B**) Enlargement of the evaluation. (**C**) If no part of the segment contains MRI signal hyperintensities of the IAN, it is considered non-visible. (**D**) If less than half to none of the segment’s volume is filled with MRI signal hyperintensities, it is considered non-visible. (**E**) If half or more than half of the segment’s volume is filled with MRI signal hyperintensities of the IAN, it is considered visible.

**Figure 3:**
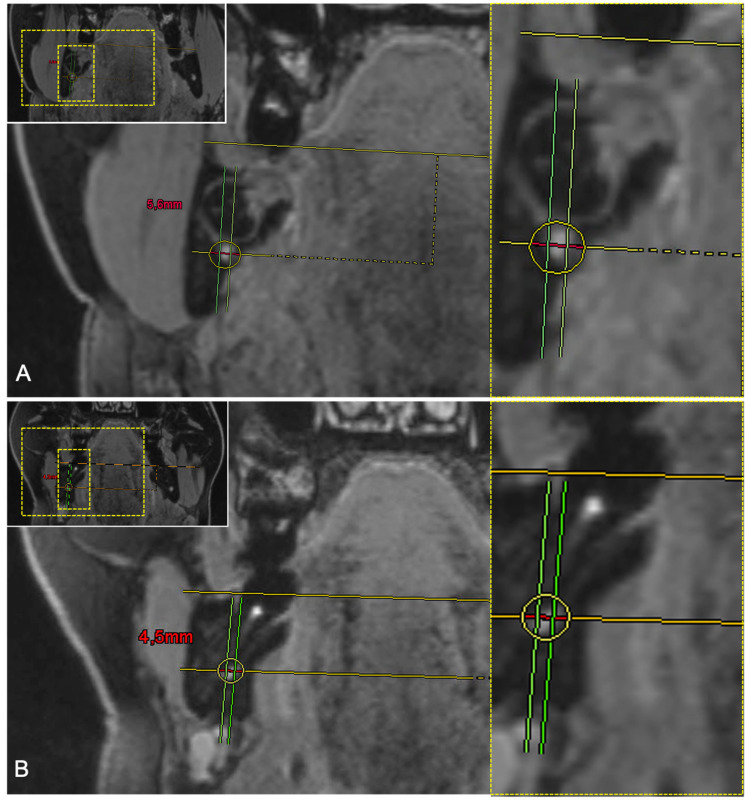
(**A**) Coronal reconstruction of a study participant’s 3D-DESS MRI images visualizing the intraosseous IAN evaluation at the third molar. For orientation, the dotted rectangles in the corner show the enlarged area. The upper middle segment (segment 2) and the lower middle segment (segment 5) are occupied by MRI signal hyperintensities. (**B**) Coronal reconstruction of a study participant’s 3D-DESS MRI images visualizing the intraosseous IAN evaluation at the level of the second molar. The upper lingual segment (segment 3), lower buccal segment (segment 4), and the lower middle segment (segment 5) are occupied by MRI signal hyperintensities.

The coronal layer of the 3D-DESS MRI reconstructions with the closest positional relationship between the IAN and the MTM, representing the clinically most relevant and dangerous zone in the surgery of impacted MTMs, was the selected reference image for the precise intraosseous IAN anatomy determination. A horizontal line was placed on the selected reference layer through the alveolar crest of the buccal cortical plate at the height of the third molar on the left and right sides. A parallel line was placed through the midpoint of the IAN, whereby the section representing the intraosseous diameter of the IAC was divided by two perpendiculars resulting in six segments: segment one, upper buccal segment; segment two, upper middle segment; segment three, upper lingual segment; segment four, lower buccal segment; segment five, lower middle segment; and segment six, lower lingual segment (Figure 1). If half or more than half of the segment’s volume is filled with MRI signal hyperintensities of the IAN, it is considered visible. However, if less than half to none of the segment’s volume is filled with MRI signal hyperintensities, it is considered non-visible (Figure 2). To investigate the influence of the impacted MTM’s position on the intraosseous IAN position, the same evaluation was performed at the distal root of the second molar (Figure 3).

#### 2.4.2. Quantitative Readout

For quantitative analysis, multiplanar reformation (MPR) and thin-slice maximum intensity projection (MIP) reconstructions of the reference layers at the impacted third molar and the distal root of the second molar were conducted. The IAN and the IAC were segmented in the 3D-DESS MRI using a semi-automated PACS contour segmentation to determine the maximum extension of the diameter in the axial, coronal, and sagittal plane.

**Figure 4:**
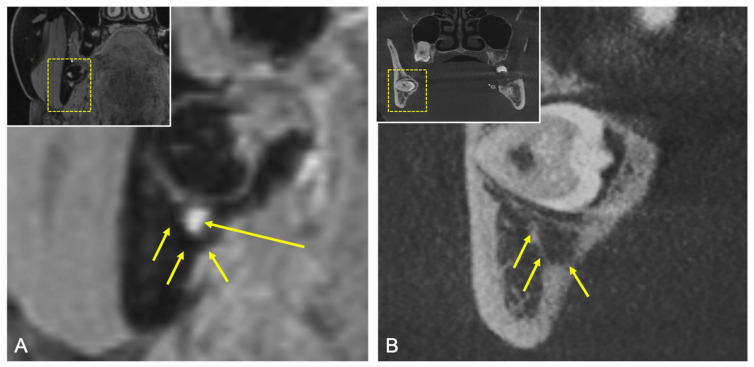
(**A**) Coronal reconstruction of the 3D-DESS MRI. For orientation, the dotted rectangles in the corner show the enlarged area. Short arrows showing the osseous boundaries of the IAC and the long arrow showing the MRI signal hyperintensities by the IAN’s tissue. (**B**) Coronal reconstruction of the CBCT. For orientation, the dotted rectangles in the corner show the enlarged area. Short arrows showing the osseous boundaries of the IAC.

Furthermore, the same contour segmentation was also performed in the same reference images at the IAC in the CBCT to discover possible correlations between the two imaging modalities (Figure 4). It was also investigated to what extent the position of the MTM influences the evaluation. Thus, the following conversion factors were determined using the MRI and CBCT imaging modalities: (1) conversion factor for IAC in CBCT and IAN in MRI and (2) conversion factor for IAC in MRI and IAN in MRI. On the one hand, the ability of MRI as an imaging modality for the osseous structures was assessed in comparison to CBCT; on the other hand, the intraosseous course of the nerve in the different planes was assessed.

### 2.5. Statistical Analysis

All statistical analyses were performed using the statistical software R 4.0.5, including the packages irr, vcd, and ggplot2. Descriptive statistics were applied for the following data evaluation. Metric variables with mean value and standard deviation were calculated. Evaluating the IAN’s intraosseous anatomical position, a percentage for each segment was calculated, indicating the probability of finding the IAN’s MRI signal hyperintensities within the specific segments. This percentage was determined based on the retention type and once independently of it in the third and second molar reference images. In the presence of a hyperintense signal in a specific segment, it was investigated whether other segments were simultaneously filled to detect certain segment combinations. This information was calculated as a percentage. For each conversion factor, the mean and standard deviation were calculated over all subjects and sides. Inferential statistics were applied to investigate whether the conversion factors between the IAC and IAN in different imaging modalities showed significant differences by performing a one-sample Wilcoxon signed-rank test. The multiple observations per subject and method were first averaged over each plane’s right and left sides, leaving only one observation per participant and imaging modality. The null hypothesis “the difference in the conversion factors in both imaging modalities is zero” was tested. If the *p*-Value was less than the specified significance level (*p* ≤ 0.05), the null hypothesis was rejected, concluding that the difference is significantly different from zero.

## 3. Results

### 3.1. Qualitative Results

**Figure 5:**
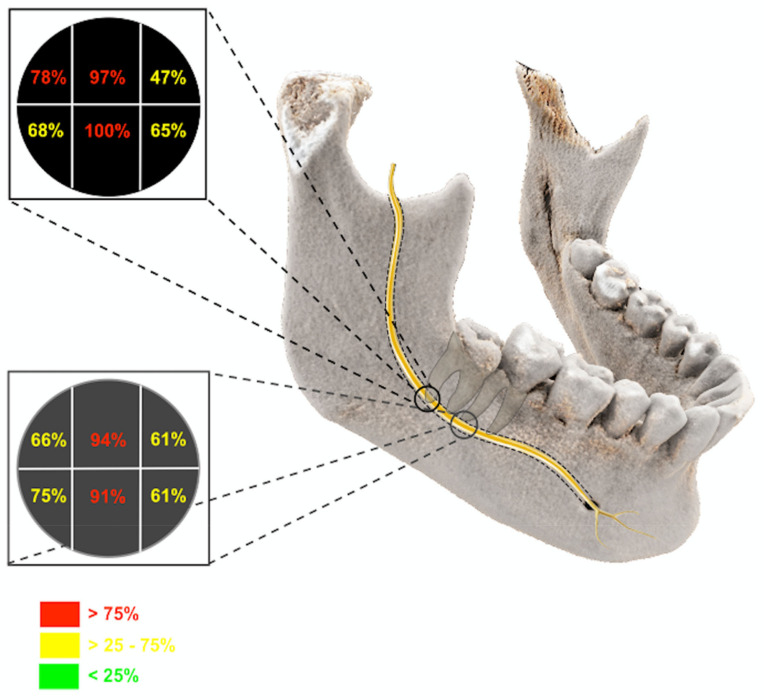
Photorealistic three-dimensional visualization of a study participant’s cone-beam computed tomography using cinematic rendering. The evaluation of the intraosseous position regardless of the positional relationship of the IAN in the reference images at the third and second molar is visualized.

The evaluation of the intraosseous IAN position regardless of the positional relationship of the IAN and MTM in the reference image at the third molar showed the highest presence of MRI signal hyperintensities in the upper middle segment (segment 2) with a percentage of 97.4%, and a percentage of 100% in the lower middle segment (segment 5) predominantly. The least detectability was registered in the upper lingual segment (segment 3, 47.4%), followed by the lower lingual segment (segment 6, 65.8%), and the lower buccal segment (segment 4, 68.4%) (Figure 5, Table 2). In the reference image at the distal root of the second molar, similar results could be observed with a percentage of 94.4% for the upper middle segment and 91.6% for the lower middle segment. The IAN’s lowest nerve tissue presence was in segments 3 and 6, with about 60% (Figure 5, Table 3). The additional examination of the IAN’s nervous tissue presence in certain segment combinations regarding the third and second molar is shown in Table 2 and Table 3.

### 3.2. Quantitative Results

**Figure 6:**
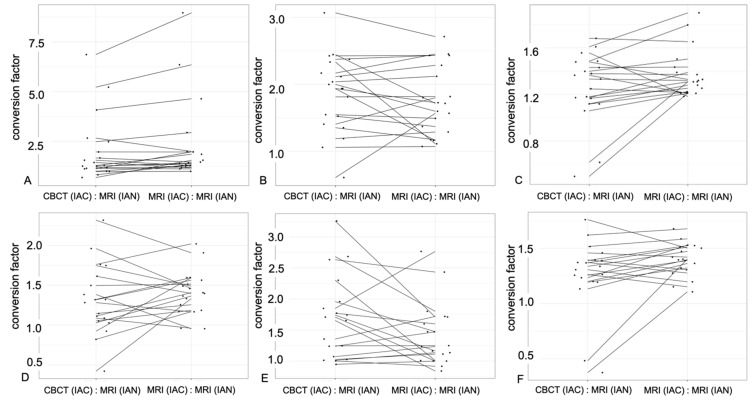
In these six plots, observations for the same subjects are connected by a line to visualize the differences between the two imaging modalities, CBCT and MRI, concerning the conversion factor in the corresponding planes. In the plots (**A–C**) (**A**, axial; **B**, sagittal; **C**, coronal), the evaluation at the third molar is visualized, and in the plots (**D–F**) (**D**, axial; **E**, sagittal; **F**, coronal), the evaluation at the level of the second molar is visualized. Thus, this graph visualizes the basis for the statistical inference using the one-sample Wilcoxon signed-rank test, which compares the difference in the two conversion factors.

At the third molar level, the mean maximal diameter expansion of the IAC in MRI was 4.18 ± 1.0 mm in the axial plane, 3.76 ± 1.1 mm in the sagittal plane, and 5.49 ± 0.8 mm in the coronal plane. In the CBCT, the diameter expansion was registered as follows: 3.9 ± 0.9 mm in the axial plane, 4.04 ± 1.2 mm in the sagittal plane, and 5.3 ± 1.0 mm in the coronal plane. The IAN in the MRI had a mean diameter expansion of 2.63 ± 1.2 mm in the axial plane, 2.38 ± 0.9 mm in the sagittal plane, and 4.12 ± 1.0 mm in the coronal plane (Table 4). In the reference image at the second molar’s distal root, the maximal diameter expansion of the IAC in MRI and CBCT and IAN in MRI registered similar mean values (Table 5). The conversion factor at the third molar between IAC in CBCT and IAC in MRI and IAN in MRI, respectively, was the following in these planes: axial (2.04 ± 2.0, 2.37 ± 2.4), sagittal (1.86 ± 1.0, 1.76 ± 0.7), and coronal (1.26 ± 0.4, 1.37 ± 0.3) (Table 4). At the level of the second molar, the values for the conversion factors were calculated as follows: axial (1.69 ± 0.9, 1.41 ± 0.4), sagittal (1.69 ± 0.9, 1.42 ± 0.7), and coronal (1.26 ± 0.4, 1.396 ± 0.2) (Table 5). At the third and second molar level, it can be stated that the conversion factor between the IAC in CBCT and IAC in MRI and IAN in MRI, respectively, showed no significant differences in all planes except the axial plane at the third and the coronal plane at the second molar (third molar: axial, *p* = 0.04; sagittal, *p* = 0.49; coronal, *p* = 0.096; second molar: axial, *p* = 0.417; sagittal, *p* = 0.167; coronal, *p* = 0. 039) (Figure 6).

## 4. Discussion

This prospective cohort study investigated the precise intraosseous localization of the IAN using a 3D-DESS MRI sequence in patients undergoing MTM surgery, showing a spatial relationship between the IAN/IAC and the MTM, indicating three-dimensional imaging. Additionally, the extent to which a conversion factor may allow for localizing the IAN’s position using the two imaging modalities CBCT and MRI was examined.

**Figure 7:**
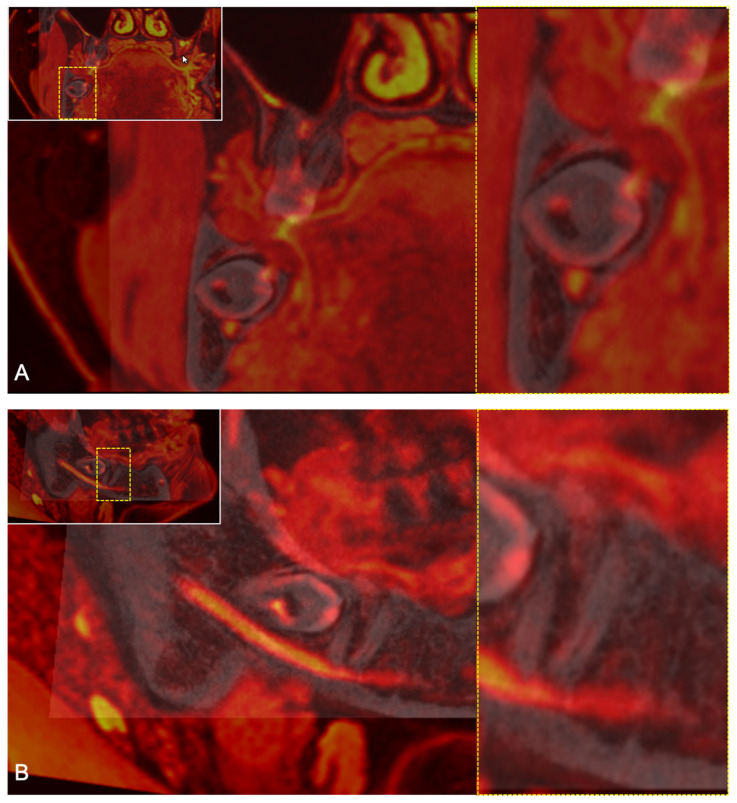
Fusion of a study participant’s 3D-DESS MRI (red) and CBCT (grey), demonstrating the good imaging quality. For orientation, the dotted rectangles in the corner show the enlarged area. (**A**) Coronal reconstruction showing the intraosseous position of the IAN (red, hyperintense signals) at the level of the third molar. (**B**) Sagittal reconstruction showing the intraosseous course of the IAN (red, hyperintense signals) at the level of the third and second molar.

A variety of MRI studies investigated the visualization of the IAN, with several reports achieving promising results of direct visualization in preoperative imaging of impacted third molars [20,21]. Data generated by the recently introduced 3D double-echo steady-state with water excitation MRI sequence provided excellent results regarding visualization of the IAN [19,23,24,25,30]. The results achieved in this study confirm the previous reports about the feasibility and accuracy of the IAN’s direct precise displaying by the 3D-DESS protocol [23] (Figure 7). This MRI protocol, with its water excitation fat-suppression technique, was originally implemented with success in everyday clinical practice for musculoskeletal imaging, localization of the parotid tumors, and visualizing the facial nerve [31]. Although there are remaining challenges in the detection of extracranial peripheral nerves, this MRI sequence presents itself as one of the most suitable modalities for displaying the IAN [23] due to the lipid-rich myelin layer surrounding its nerve axons. The remaining challenges in detecting extracranial peripheral nerves include the nerve’s small dimension and the distinguishment from proximal and partially overlapping anatomical structures.

Regarding the precise anatomical intraosseous IAN position, the central part of the nerve canal is nearly always filled by the IAN’s nerve tissue at the third and second molar level, irrespective of the positional relationship to the MTM. Furthermore, there is a tendency that if one of the lingual segments (segment 3 or 6) contains MRI signal hyperintensities, the other lingual IAC segment is simultaneously filled by nerve tissue. The same statement can also be applied to the buccal segments (segment 1 or 3). Additionally, it can be stated that in cases where the IAN’s intraosseous position is located buccally, it can be assumed that the lingual segments of the IAC are frequently not filled with IAN tissue. Comparable findings regarding the lingual upper segment (segment 3) could be stated at the second molar region, with a certain tendency for the IAN’s intraosseous position not to fill the buccal portion of the IAC in about 30% of the cases.

The retention type of the third molar tooth seems to influence the intraosseous position of the nerve, as the data of this study show that the root of the third molar often displaces the IAN in the segments proximal to the contact site. At the height of the second molar, the intraosseous position showed a more variable distribution, demonstrating tendencies that the IAN tissues probably fill the whole volume of the IAC. In the non-contact cases, it is challenging to describe trends. Due to the small number of cases and the study population, these statements should all be treated with caution. To the best of the authors’ knowledge, this is the first study investigating the IAN’s intraosseous position by direct visualization using MRI. Previously conducted studies documenting the intraosseous course of the IAN had some limitations, such as the use of only CBCT imaging displaying the nerve canal [26], the use of human cadavers showing the nerve plexus by dissections without any references to third molars [32], or other skull studies demonstrating the intraosseous branching of the IAN in edentulous mandibles [27,28] not allowing for generally valid statements. In the evaluated MRI images, the osseous boundaries of the nerve canal and the intraosseous neural tissue could be visualized in all cases, which enabled the intraosseous location of the IAN. Moreover, several studies state that the IAC in the molar region has an even higher detectability in MRI compared to CT [33] and CBCT [34]. Nevertheless, it should be mentioned that the standard procedure for imaging bone tissue is still CT, and specifically CBCT, in the dentomaxillofacial field. However, the accuracy of displaying the IAC’s osseous cortical boundaries by conventional radiation-based three-dimensional imaging modalities can be affected by the bone’s quality and density [35], patient-related factors such as abnormal position and angulation of the MTM’s root morphology, or special cases such as concrescence of third and fourth supernumerary impacted MTMs [36]. Among these particular circumstances, CBCT remains the gold standard for the visualization of osseous structures. The weighting of the importance of these individual factors before surgery remains difficult. Although it has to be mentioned that these factors and others, such as bone thickness, level of sclerosis around the MTM, and morphology of the roots, are assessed along with the localization of the nerve prior to the surgical intervention. These data can be effectively assessed in CT and CBCT and have not been investigated in MRI. Nevertheless, recent findings show the superiority of MRI in the visualization of inflammatory processes of soft tissues [37]. Further, the use of CBCT shows difficulties in visualizing the IAC in the first molar region in cases where a clear-cut bony delimitation of the IAC is detectable [33]. In these specific cases, and since CBCT cannot directly visualize the IAN, MRI remains the only imaging modality that allows for the direct localization of the nerve and could be indicated to decrease the underestimation of the nerve’s expansion and, thus, its injury in surgical interventions performed near the IAC.

MRI imaging using the 3D-DESS sequence brings the advantages of high-resolution and high-contrast images, allowing for simultaneous visualization of the IAC’s osseous boundaries and the IAN’s nerve tissue, enabling reliable assessment of the IAN’s course in relation to the third molars [30] and relatively short acquisition times [23]. Furthermore, MRI avoids X-ray exposure to the patient, which could reduce the increasing incidence of cancer due to CBCT application in the preoperative management of third molar surgery [38].

However, there are remaining challenges, such as the high costs and the globally reduced accessibility at present [39]. For these reasons, both MRI and CT/CBCT imaging will remain a part of future clinical routines. Our study attempted to discover a conversion factor in each plane between the two imaging modalities CBCT and MRI, aiding in the localization of the intraosseous IAN position. The conversion factors between the IAN in MRI and the IAC in MRI and the CBCT, respectively, showed no significant differences except the axial plane at the third and the coronal plane at the second molar, confirming the statement that the visualization of the IAC’s osseous boundaries is feasible and accurate for both imaging modalities. However, the significant differences in these two conversion factors can be explained due to chance and should be interpreted with caution because the difference might be clinically irrelevant.

Despite the large inter-variability in terms of positional relationship to adjacent anatomical structures and various intraosseous branching, bilateral symmetry was observed between the right and left sides [32]. Besides, the intraosseous buccolingual IAC location was shown to be dependent on age and race [26]. There are no other studies investigating conversion factors between the two implemented imaging modalities to the authors’ knowledge. However, due to specialized tools such as mandibular coils [16,17] or mobile bedside MRI scanners, conversion factors may play an essential role in the future, which is why standardization should be considered.

Besides the already mentioned application in the daily clinical routine in musculoskeletal radiology, in parotid tumor diagnostics, and the associated visualization of the facial nerve, the DESS sequence can help us in various other complex surgical interventions in the oral cavity where osteotomy close to sensitive soft tissue might be necessary.

By a further refinement of this MRI sequence, it might be helpful in maxillary sinus elevation surgery [40,41], salivary gland diagnostics [42,43], or might be used in superimposing with other digital images such as intraoral scans in guided implant surgery.

From a clinical perspective, CBCT will continue to be an integral part of the preoperative radiological assessment in MTM surgery. The targeted use of Dental MRI in deeply impacted high-risk cases with an unclear spatial relationship between the IAN and MTM might provide additional diagnostic information compared to the use of CBCT only. Black Bone MRI Sequences seem to have no significant limitations in diagnostic information and even provide superior diagnostic information in displaying inflammatory processes in the MTM region. Nevertheless, prospective studies are required to assess these findings’ therapeutic and clinical impact using direct MRI visualization.

This study has some limitations regarding the methodology. First, the small sample size does not allow for generally valid statements; therefore, further studies need to investigate larger cohorts to confirm the trends obtained with high reliability and validity, allowing for ideal sample size calculation. Second, the subjective evaluation of the IAN could be influenced by the evaluator’s bias or by various systematic biases. It is preferable to supplement or partially replace this evaluation method with more objective evaluation options, generating a more reliable evaluation. Third, most of the study participants rarely showed artifacts due to dental restorations. In addition, all patients who had implants near the first and second mandibular molar were excluded from the study. Thus, the MRI sequence should be refined using zero TE or ultra-short TE methods, which could minimize these artifacts.

This radiation-free non-invasive imaging modality allows for the preoperative determination of the intraosseous IAN position. The direct visualization provides an advantage in all cases where CBCT shows an overlap of the nerve canal by the third molar roots. However, the question of the extent to which the nerve is displaced remains unclear. To optimize the individual treatment of these patients, the use of the 3D-DESS MRI protocol may lead to an improvement.

## 5. Conclusions

This 3D-DESS MRI protocol demonstrates good feasibility of accurate simultaneous visualization of osseous structures and nerve tissue, providing an excellent radiation-free imaging modality for preoperative determination of the IAN’s intraosseous anatomical position in the management of difficult MTM surgery. In the future, the targeted use of 3D-DESS MRI might be beneficial in the individual treatment of anatomically complex situations with deeply impacted MTMs with fully developed roots close to IACs and incomplete structural integrity of the osseous boundaries.

## Figures and Tables

**Table 1:** Patient characteristics.

Patient Characteristics	Total
N	19
Gender, male/female, N	6/13
Mean (SD) age at scan, years	30.5 (13)
Median age at scan, years	25
Age range, years	18–63
Totally evaluated Inferior alveolar nerves	36
Clinical indication	MTM Surgery
Retention types [29]	
Type 1, N	0
Type 2, N	0
Type 3, N	11
Type 4, N	19
Type 5, N	2
Type 6, N	0
Type 7, N	0
No retention	4
Absent	2

**Table 2:** Percentage of the IAN’s intraosseous position regardless of the positional relationship of the IAN and third molar at the level of the third molar. In the presence of a hyperintense signal in a specific segment, it is noted whether other segments were simultaneously filled.

Third Molar	Segment 1	Segment 2	Segment 3	Segment 4	Segment 5	Segment 6
Generally Yes	78.9%	97.4%	47.4%	68.4%	100%	65.8%
If Segment 1 Yes	-	93.3%	53.3%	70%	96%	63.3%
If Segment 2 Yes	78.4%	-	48.7%	67.6%	100%	67.6%
If Segment 3 Yes	88.9%	100%	-	61.1%	100%	100%
If Segment 4 Yes	88.5%	96.2%	46.2%	-	100%	69.2%
If Segment 5 Yes	78.9%	97.4%	52.6%	65.8%	-	65.8%
If Segment 6 Yes	84%	100%	72%	72%	100%	-

**Table 3:** Percentage of the IAN’s intraosseous position at the level of the second molar. In the presence of a hyperintense signal in a specific segment, it is noted whether other segments were simultaneously filled.

Second Molar	Segment 1	Segment 2	Segment 3	Segment 4	Segment 5	Segment 6
Generally Yes	66.7%	94.4%	61%	75%	91.6%	61.1%
If Segment 1 Yes	-	95.8%	62.5%	95.8%	95.8%	58.3%
If Segment 2 Yes	67.6%	-	73.5%	73.5%	88.2%	64.7%
If Segment 3 Yes	63.6%	100%	-	72.7%	90.9%	81.8%
If Segment 4 Yes	85.2%	96.3%	59.3%	-	96.3%	66.7%
If Segment 5 Yes	66.7%	90.9%	63.6%	75.8%	-	66.7%
If Segment 6 Yes	68.2%	100%	81%	77.3%	100%	-

**Table 4:** The maximal diameter expansion of the IAC in CBCT and in MRI and the IAN in MRI was determined in the axial, sagittal, and coronal plane at the level of the third molar. A conversion factor between IAC and IAN using the two imaging modalities was determined.

Third Molar	Axial	Sagittal	Coronal
IAC in CBCT	3.9 ± 0.85 mm	4.04 ± 1.23 mm	5.3 ± 1.03 mm
IAC in MRI	4.18 ± 0.97 mm	3.76 ± 1.1 mm	5.49 ± 0.83 mm
IAN in MRI	2.63 ± 1.19 mm	2.38 ± 0.89 mm	4.12 ± 0.98 mm
Conversion factor IAC (CBCT): IAN (MRI)	2.04 ± 1.953	1.86 ± 0.96	1.258 ± 0.394
Conversion factor IAC (MRI): IAN (MRI)	2.367 ± 2.413	1.755 ± 0.742	1.374 ± 0.252

**Table 5:** The maximal diameter expansion of the IAC in CBCT and in MRI and the IAN in MRI was determined in the axial, sagittal, and coronal plane at the level of the second molar. A conversion factor between IAC and IAN using the two imaging modalities was determined.

Second Molar	Axial	Sagittal	Coronal
IAC in CBCT	4.22 ± 0.78 mm	3.86 ± 0.9 mm	5.28 ± 0.83 mm
IAC in MRI	4.23 ± 1.21 mm	3.09 ± 1.09 mm	5.62 ± 0.87 mm
IAN in MRI	3.13 ± 0.89 mm	2.4 ± 0.8 mm	4.1 ± 0.78 mm
Conversion factor IAC (CBCT): IAN (MRI)	1.692 ± 0.864	1.691 ± 0.864	1.258 ± 0.407
Conversion factor IAC (MRI): IAN (MRI)	1.407 ± 0.427	1.424± 0.727	1.396 ± 0.223

## Data Availability

The data presented in this study are available on request from the corresponding author. The data are not publicly available due to privacy restrictions.
